# Correction to: ‘Coral reef fish smell leaves to find island homes’ (2022) by Dixson *et al.*

**DOI:** 10.1098/rspb.2022.1243

**Published:** 2022-12-21

**Authors:** Danielle L. Dixson, Geoffrey P. Jones, Philip L. Munday, Serge Planes, Morgan S. Pratchett, Maya Srinivasan, Craig Syms, Simon R. Thorrold

*Proc. R. Soc. B*. **275**, 2831–2899. (Published online 26 August 2008). (https://doi.org/10.1098/rspb.2008.0876)

Due to an error in their calculation, the standard errors reported in table 1 of the article [1] were too small. Please see the revised table 1 for correct s.e. Correction of this error does not change the conclusions of our work.

**Table 1.** Results of pairwise olfactory choice experiments on field-collected juvenile *A. percula*, including the choices made between (*a*) water from reefs with and without islands, (*b*) water from different distances away from islands and (*c*) water with and without anemones and rainforest leaves. In addition, (*d*) shows the pairwise trials for laboratory-reared juveniles and their choices between water with and without anemones and leaves. Data are mean percentage of time spent in water flowing from the two sources ± s.e. Statistic tests are *χ*^2^-tests on the number of trials where larvae exhibited a preference for one water source in the test chamber. *n*, sample size; *p*, probability of the data given the null hypothesis that there is no choice.

pairwise test	choice 1, mean % time ± s.e.		choice 2, mean % time ± s.e.		*χ*^2^	*n*	*p*
(*a*) field experiment 1: reefs with and without islands	Tuare Is.	99.6 ± 0.19	South Bay Reef	0.4 ± 0.19	28.0	28	<0.001
	Kimbe Is.	99.5 ± 0.23	May Reef	0.5 ± 0.23	22.0	22	<0.001
(*b*) field experiment 2: distance from island	Tuare Is. beach	98.0 ± 0.67	Tuare Is. offshore	2.0 ± 0.67	22.0	22	<0.001
	Tuare Is. beach	95.0 ± 0.61	Tuare Is. crest	5.0 ± 0.61	22.0	22	<0.001
	Tuare Is. crest	55.0 ± 1.14	Tuare Is. offshore	45.0 ± 1.14	0.17	22	0.683
	Kimbe Is. beach	93.0 ± 1.59	Kimbe Is. Offshore	7.0 ± 1.59	24.0	24	<0.001
	Kimbe Is. beach	97.2 ± 0.60	Kimbe Is. crest	2.8 ± 0.60	20.2	24	<0.001
	Kimbe Is. crest	57.0 ± 1.66	Kimbe Is. Offshore	43.0 ± 1.66	0.0	24	1
(*c*) field experiment 3: response to anemones and leaves	anemone	91.0 ± 1.41	no anemone	9.0 ± 1.41	18.2	22	<0.001
	mixed leaves	89.5 ± 1.74	no leaves	10.5 ± 1.74	20.2	24	<0.001
	leaves sp. 1	90.0 ± 1.68	no leaves	10.0 ± 1.68	20.0	20	<0.001
	leaves sp. 2	92.0 ± 1.06	no leaves	8.0 ± 1.06	20.0	20	<0.001
	leaves sp. 3	90.0 ± 1.24	no leaves	10.0 ± 1.24	16.2	20	<0.001
	leaves sp. 4	92.0 ± 1.45	no leaves	8.0 ± 1.45	20.0	20	<0.001
	leaves sp. 5	94.0 ± 1.02	no leaves	6.0 ± 1.02	20.0	20	<0.001
(*d*) laboratory experiment: response of naive larvae to anemones and leaves	anemone	98.0 ± 0.44	no anemone	2.0 ± 0.44	24.0	24	<0.001
	rainforest leaves	96.0 ± 0.45	no leaves	4.0 ± 0.45	36.0	36	<0.001
	melaleuca leaves	0.0 ± 0.0	no leaves	100.0 ± 0.0	30.0	30	<0.001

Additionally, we would like to clarify that the strong treatment effects found in this experiment are likely the result of a combination of factors including, but not limited to, the ecology of the focal species, the flume apparatus, the chemical comparisons being tested as well as the concentration of the chemical cues tested. The chemical cue concentration is likely higher than the organisms would experience in nature and therefore likely a form of supernormal stimuli. Simply put, supernormal stimuli are bigger and more intense than normal, and elicit a larger than normal response from the animal [2]. Here, the naturally occurring olfactory cues indicate habitat; the heightened preference when the stimulus is offered at an intense concentration follows this behavioural pattern. The research presented in this study purposefully used strong chemical cues to determine *if* chemical cues are used in habitat selection, rather than determining a detection threshold for this species of a concentration gradient.
